# Causal relationship between outdoor atmospheric quality and pediatric asthma visits in hangzhou

**DOI:** 10.1016/j.heliyon.2023.e14271

**Published:** 2023-03-07

**Authors:** Yuqing Feng, Yingshuo Wang, Lei Wu, Qiang Shu, Haomin Li, Xin Yang

**Affiliations:** a Department of Data and Information, Children's Hospital, Zhejiang University School of Medicine, National Clinical Research Center for Child Health, National Children's Regional Medical Center, Hangzhou, 310052, China; b Department of Pulmonology, Children's Hospital, Zhejiang University School of Medicine, National Clinical Research Center for Child Health, National Children's Regional Medical Center, Hangzhou, 310052, China; c Department of Endoscopy Center, Children's Hospital, Zhejiang University School of Medicine, National Clinical Research Center for Child Health, National Children's Regional Medical Center, Hangzhou, 310052, China; d Department of Genetics and Metabolism, Children's Hospital, Zhejiang University School of Medicine, National Clinical Research Center for Child Health, National Children's Regional Medical Center, Hangzhou, 310052, China

**Keywords:** Children, Asthma, Meteorological variables, Air pollutants, Causality, Asthma patient visits

## Abstract

Many air pollutants and climate variables have proven to be significantly associated with pediatric asthma and have worsened asthma symptoms. However, their exact causal effects remain unclear. We explored the causality between air pollutants, climate, and daily pediatric asthma patient visits with a short-term lag effect. Based on eight years of daily environmental data and daily pediatric asthma patient visits, Spearman correlation analysis was used to select the air pollutants and climate variables that correlated with daily pediatric asthma patient visits at any time (with a lag of 1–6 days). We regarded these environmental variables as treatments and built multiple- and single-treatment causal inference models using the Dowhy library (a Python library for causal inference by graphing the model, quantitatively evaluating causal effects, and validating the causal assumptions) to estimate the quantitative causal effect between these correlated variables and daily pediatric asthma patient visits in lag time. The multiple-treatment causal inference model was a model with 8 treatments (Visibility, Precipitation, PM_10_, PM_2.5_, SO_2_, NO_2_, AQI and CO), 1 outcome (daily pediatric asthma patients visits), and 5 confounders (Humidity, Temperature, Sea level pressure, wind speed and unobserved confounders “U”). Single-treatment causal inference models were 8 models, and each model has 1 treatment, 1 outcome and 12 confounders. Spearman correlation analysis showed that precipitation, wind speed, visibility, air quality index, PM_2.5_, PM_10_, SO_2_, NO_2_, and CO were significantly associated variables at all times (p < 0.05). The multiple-treatment model showed that pooled treatments had significant causality for the short-term lag (lag1–lag6; p < 0.05). Causality was mainly due to SO_2_. In the single-treatment models, visibility, SO_2_, NO_2_, and CO exhibited significant causal effects at any one time (p < 0.05). SO_2_ and CO exhibited stronger positive causal effects. The causal effect of SO_2_ reached its maxima (causal effect = 11.41, p < 0.05) at lag5. The greatest causal effect of CO appeared at lag3 (causal effect = 10.67, p < 0.05). During the eight year-period, the improvements in SO_2_, CO, and NO_2_ in Hangzhou were estimated to reduce asthma visits by 8478.03, 3131.08, and 1341.39 per year, respectively. SO_2_, NO_2_, CO, and visibility exhibited causal effects on daily pediatric asthma patient visits; SO_2_ was the most crucial causative variable with a relatively higher causal effect, followed by CO. Improvements in atmospheric quality in the Hangzhou area have effectively reduced the incidence of asthma.

## 1 Introduction

Asthma is defined as ‘a chronic inflammatory disorder of the airways in which many cells play a role, including mast cells and eosinophils’, according to the International Consensus Report on the Diagnosis and Treatment of Asthma [1]. It is a common chronic respiratory disease that affects over 300 million people globally [2], and it has a higher prevalence in children than in adults [3].Several existing cross-sectional or cohort studies have suggested that in the past 40 years, the prevalence of pediatric asthma has increased in many countries or regions [4]. China is among the most asthma-afflicted countries. According to data released in 2013, the prevalence of pediatric asthma in China has been increasing over the past decades, reaching approximately 10 million children; the prevalence of pediatric asthma in different areas of the country was 2.0–4.2% [5,6]. Pediatric asthma control in China remains a challenge despite the large number of patients present. Approximately 20% of uncontrolled pediatric asthma patients in China [7], which seriously affects children's health and increases the financial burden on families and society. Therefore, it is necessary to explore the etiology of asthma and to assist parents, doctors, and public health agencies in preventing and controlling asthma in children.

To date, abundant evidence has shown that several air pollutants and meteorological variables have exogenous effects on asthma attacks. Specifically, PM_2.5_ was shown to be positively correlated with pediatric asthma prevalence with a short-term lag effect [8,9]; temperature changes between neighboring days had a positive association with the risk of pediatric asthma exacerbation in warm seasons (from May to October) [10], and relative humidity and hours of sunshine were correlated with meteorological parameters, with respect to the prevalence of pediatric asthma [5]. However, these studies have only demonstrated the positive or negative associations of these variables with pediatric asthma. Their correlations may also be influenced by other variables, such as interactions between variables and other unobserved confounders. Based on these findings, we did not know whether these variables were the direct cause of the disease, and we did not know that if we improved the quality of the atmosphere, the number of childhood asthma visits would reduce or not. This also means that while correlation can aid prediction, it is not valid evidence to aid decision-making. Theoretically, in studies aimed at assisting decision making, the correctness of the underlying mechanism (the direction of causality and quantitative causal effect) is more important, and differences between them can lead to different conclusions. Thus, this study aimed to determine the correct underlying mechanism by separately exploring the causality of environmental variables (air pollution variables and meteorological variables) on daily pediatric asthma patient visits (DPAPVs) and the effect of the short-term lag on causality in Hangzhou, China from 2014 to 2021. Then, we can provide effective recommendations for preventing and controlling pediatric asthma.

## 2 Materials and methods

### 2.1 Meteorological data and air pollution data

**Fig. 1:**
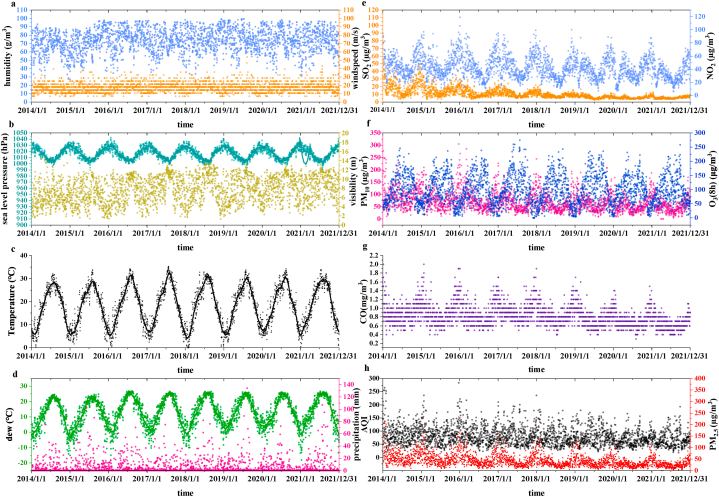
Climate and Air pollution data during 2014 and 2021 at the study site. a. humidity and windspeed; b. Sea level pressure and visibility; c. Temperature; d. Dew and precipitation; e. SO_2_ and NO_2_; f. PM_10_ and O_3_; g. CO; h. AQI and PM_2.5_.

Hangzhou is located in southeastern China in the subtropical monsoon zone and has four distinct seasons. In this study, both the daily mean meteorological data and daily mean air pollution data were obtained from January 1, 2014 to December 31, 2021 (detailed distributions of meteorological data and air pollution data are shown in Fig. 1a–h, respectively). All concentrations of outdoor air pollutants were lower than the levels of ambient air quality standards in China (GB 3095–2012). The daily mean meteorological data (temperature, feels-like temperature, dew, humidity, precipitation, wind speed, visibility, and sea level pressure) were downloaded from the National Center for Environmental Information (https://www.ncei.noaa.gov), which is an agency of the United States government that manages one of the world's largest archives of atmospheric data. The data obtained were vetted using standards established by the National Research Council (NRC). Daily air pollution data [24-h mean concentrations of air quality index (AQI), PM_2.5_, PM_10_, SO_2_, NO_2_, CO, and daily maximum 8-h mean concentrations of O_3_] were obtained from the national air pollution prevention and control monitoring network, which includes 1436 monitoring stations in 338 cities in China (there are 24 monitoring stations in Hangzhou). Both daily mean meteorological data and daily mean air pollution data were complete.

### 2.2 Asthma patient data

From January 1, 2014 to December 31, 2021, records of children with an outpatient or emergency diagnosis of asthma were collected from the Children's Hospital Affiliated to Zhejiang University School of Medicine, which is located in Hangzhou. It is the center of children's health care in Zhejiang Province and the National Clinical Research Center for Children's Health and Disease in China. In this study, pediatric asthma was defined based on age <18 years and the ICD-10 code J45. This study focused on the first visit after a particular asthma attack, scheduled follow-up visits within a week (and a month) and repeated visits within a day were excluded from the patient dataset, with a final total of 217,950 visits recorded a diagnosis of asthma. This study was approved by the Institutional Review Board of the Children's Hospital of Zhejiang University School of Medicine (2018-IRB-046), and the requirement for informed consent was waived.

### 2.3 Statistical methods

#### 2.3.1 Primary analysis

Descriptive statistics calculated using R (version 4.0.2) were used to summarize the general statistical characteristics of the data included in this study. For descriptive statistics, the patient age (median [Q1, Q3]), sex (female and male, %), and four seasons (from March to May is spring, from June to August is summer, from September to November is autumn, and from December to February is winter, %) were calculated. The Shapiro‒Wilk test was used to explore the non-normal distribution of the data. Associations between environmental variables (air pollutant concentrations and meteorological variables) and DPAPVs were assessed using the Spearman correlation coefficient in R, and a p-value below 0.05 was regarded as statistically significant. Associations between the current day (lag0) and the previous 1–6 days (lag1 to lag6) were calculated, which means each environmental variable and DPAPVs had 7 calculated associations (lag0 to lag6). Because correlation is a necessary but not sufficient condition for causation, an environmental variable that had a significant association at any time (lag0 to lag6) was considered a highly related environmental variable and was used to explore the causalities.

#### 2.3.2 Causality analysis by dowhy

The Dowhy library is a Python library for causal inference that graphs the model, quantitatively evaluates causal effects, and validates the causal assumptions. It has a state-of-the-art application programming interface (API) automatically validates the causal assumptions for any estimation method in order to complete the refutation [11]. In this study, the causal inference model of the Dowhy library was used to assess the causality between the associated environmental variables and the DPAPVs. Supplementary Fig. S1 shows the flow of the Dowhy causal-inference model. Initially, we assumed that the treatment was a causal variable for the DPAPVs. Four key steps were then implemented: modeling, identification, estimation, and refutation.

●Modeling: A causal graph was encoded and created. In this graph, the aim environmental variable was the treatment, the DPAPVs were the outcome, the remaining variables were confounders, and the variable U represented unobserved confounders (such as medication status, pollen concentration) which may influence causal relationships between environmental variables and outcome but not collected in this study.
●Identification: The effect of treatment was identified by the “backdoor criterion” [12].This criterion was used to cut off the back paths (they present the correlation instead of causality between treatment and outcome) in causal inference model.
●Estimation: The identified effect of the treatment was estimated by the Poisson method, and a test p-value <0.05 was regarded as statistically significant.
●Refutation: Three methods (adding a random common cause, use of a placebo treatment, and use of a subset of data) were used to validate the results of the estimation step.

In modeling step, the causal inference model of the Dowhy library creates the causal graph with treatment, outcome, and confounders. Then, in identification and estimation steps, the model will estimate the effect of the treatment after adjusting the effect of confounders. In the refute step, the “random common cause” approach implies that a synthetic independent random variable is added to the original data to verify the sensitivity of the model to unobserved confounding variables. The “placebo treatment” approach is similar to cross-validation in predictive models. It replaces the real treatment with a random independent variable to test whether the entire model contains a mistake (with the known causal effect of the variable). In the “subset of data” approach, the original data are replaced by randomly selected subsets to evaluate the variance of the effect generated in the evaluation step [11]. When the results of the ‘‘random common cause’’ approach do not significantly vary with this estimated effect, the same happens with the ‘‘subset of data’’ approach, and the result of the “placebo treatment” approach is close to 0 at the same time, which means that the estimator is robust, i.e., the estimated effect passes the three validation approaches, and the treatment is the causal variable of the disease.

As is understood currently interactions between treatments and indirect causes of outcomes will influence treatment causality. Therefore, we assessed the causal effect of each highly related environmental variable by comparing multiple- and single-treatment causal inference models (with or without indirect causes). First, we regarded all highly related environmental variables as treatments and estimated the pooled causal effect of treatments with interactions using a multiple-treatment causal inference model. Second, we used the single-treatment causal inference model to prove the causality of each variable with or without indirect causes. The term “indirect causes” meant that there was a causal relationship with treatments instead of the outcome (DPAPVs). Finally, we compared the results of the three types of models to prove the causality of each variable and the influence of interactions and indirect causes. In addition to the effect of interactions and indirect causes, this study explored the short-term lag effect of each variable's causality.

## 3 Results

### 3.1 Characteristics of the study cohort

The Shapiro-Wilk test of the daily patient visits (p < 0.01) determined the non-normal distribution of patient data. Descriptive statistics for the number of pediatric asthma patients are summarized in Table 1.

**Table 1:** Summary statistics of pediatric asthma patient visits.

Characteristics	Values
**Age median [Q1, Q3] (years)**	5 [4,8]
**Sex (%)**	
**Female**	76,978 (35.32%)
**Male**	140,972 (64.68%)
**Season (%)**	
**Spring**	44,869 (20.59%)
**Summer**	51,098 (23.44%)
**Autumn**	54,178 (24.86%)
**Winter**	67,805 (31.11%)
**Year (%)**	
**2014**	34,306 (15.74%)
**2015**	32,866 (15.08%)
**2016**	33,889 (15.55%)
**2017**	30,433 (13.96%)
**2018**	28,799 (13.21%)
**2019**	25,417 (11.66%)
**2020**	14,779 (6.78%)
**2021**	17,461 (8.01%)
**Total**	217,950

The total number of DPAPVs was 217,950. The mean age at visits was 5.67 years with an interquartile range (IQR) of [4,8]. In the sex subgroups, the number of males (140,972, 64.68%) was approximately one-fold higher than that of females (76,978, 35.32%). Among the four seasons, the number of visits in spring was the lowest (44,869, 20.59%), followed by that in summer (51,098, 23.44%). Approximately one-third of the total number of patient visits occurred during the winter (67,805, 31.11%). The annual number of asthma visits has shown an overall decreasing trend, except from 2020 to 2021.

### 3.2 Correlations between the environmental variables and the DPAPVs for the short-term lag

**Fig. 2:**
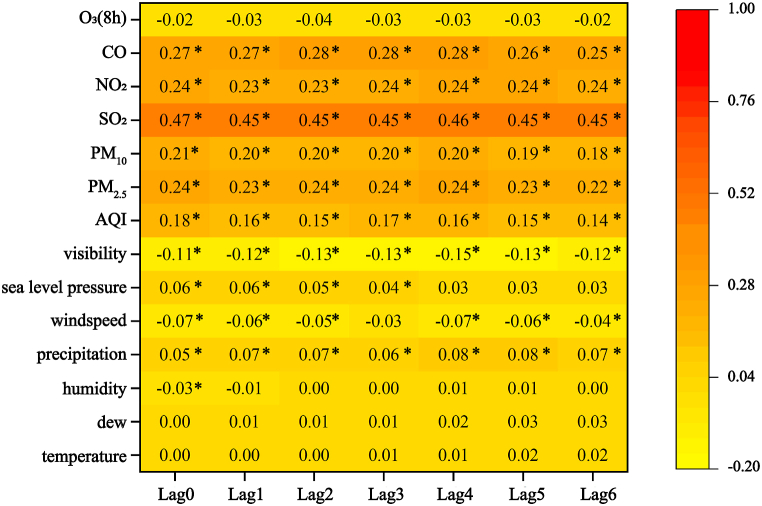
The short-term lag effect of the Spearman correlation analysis between environmental VARIABLES and daily pediatric asthma patient visits (* indicated the correlation coefficient is statistically significant at p < 0.05).

The Spearman correlation coefficients describing the relationships between the DPAPVs and environmental variables (air pollutants and meteorological variables) are shown in Fig. 2, where rs is the Spearman's correlation coefficient. As shown in Fig. 2, precipitation, visibility, AQI, PM_2.5_, PM_10_, SO_2_, NO_2_, and CO were significantly associated with the DPAPVs (p < 0.05) from lag0 to lag6. Among these variables, visibility was the only variable that had negative associations (rs<0) with the DPAPVs for the short-term lag. In addition, air pollutants generally had stronger correlations with DPAPVs, compared to those with meteorological variables. Among these associated air pollutants, the number of DPAPVs was most closely related to SO_2_, followed by CO; their maximum correlations appeared in lag0 (rs=0.47,p<0.05) and lag2–lag4 (rs=0.28,p<0.05), respectively.

In general, precipitation, wind speed, visibility, AQI, PM_2.5_, PM_10_, SO_2_, NO_2_, and CO were the highly associated variables, with significant correlations from lag0 to lag6. Moreover, compared to other variables, SO_2_ and CO had relatively stronger correlations with the DPAPVs. The Spearman correlation coefficients of the air pollutants and meteorological variables are shown in Supplementary Fig. S2 and indicated that most environmental variables had moderately high correlation coefficients with each other and that air pollutants generally had negative correlations with meteorological variables.

### 3.3 Causality inference between the environmental variables and DPAPVs in short-term lag

#### 3.3.1 Causality inference with multiple treatments in short-term lag

**Fig. 3:**
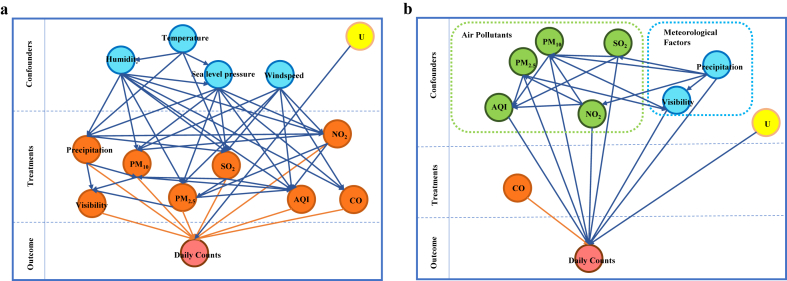
Causal inference models a. The multiple treatments causal inference model; b. The single treatment causal inference model with direct causal variables.

The increase in visits for patients with asthma was caused by multiple reasons in real life. Therefore, we used the multiple-treatment causal inference model to explore the pooled causality of these variables. Fig. 3a shows the multiple-treatment causal inference model used in this study. The explored highly associated environmental variables (visibility, precipitation, AQI, PM_2.5_, PM_10_, SO_2_, NO_2_, and CO) were treatments in this causal inference model, as indicated by orange circles, and the proven causal relationships are indicated by blue arrows. These relationships included the settling effect of precipitation and humidity, the diffusion effect of the wind speed, the catalytic effect of temperature, the mutual transformation of air pollutants, and the interaction between meteorological variables and air pollutants [13,14]. Unproven causal associations included potential causal relationships between DPAPVs and the different treatments. After building the multiple-treatment causal inference model, the effects of the pooled and each variable were estimated.

The estimated effect of pooled treatments and each treatment in the multiple-treatment causal inference model is shown in Table 2. This indicates that there was a change in outcome when the treatment was increased by one unit. The results indicated that pooled treatments had significant estimated effects (p < 0.05) on the daily patients’ attendance only in lag time (lag1 to lag6), which suggested that pooled treatments had significant causalities on the DPAPVs with short-term lag effects. Moreover, only SO_2_ had a significant estimated effect on lag time, and its value was close to that of the pooled treatments. However, the estimated effects may be influenced by different daily fluctuations, units, interactions between treatments, and indirect variables (humidity, temperature, sea level pressure, and wind speed). Therefore, the results of the multiple-treatment causal inference model only proved that these variables would cause DPAPVs with a short-term lag effect, and this meant that SO_2_ provided most of the causality associated with the pooled treatments.

**Table 2:** The estimated effect of pooled treatments and each treatment in multiple treatments causal inference model from lag0 to lag6.

	Lag0	Lag1	Lag2	Lag3	Lag4	Lag5	Lag6
**Pooled**	0.29	**1.02**	**0.97**	**0.94**	**1.00**	**1.12**	**1.10**
**Precipitation (mm)**	**0.02**	0.04	0.05	0.03	0.06	0.06	0.06
**Visibility(m)**	**0.24**	0.10	0.05	0.05	−0.01	0.02	−0.02
**AQI**	**0.00**	0.02	0.02	0.03	0.03	0.03	0.04
**PM**_**2.5**_**(μg/m**^**3**^**)**	**0.00**	−0.14	−0.15	−0.15	−0.10	−0.08	−0.13
**PM**_**10**_**(μg/m**^**3**^**)**	**−0.01**	−0.05	−0.05	−0.06	−0.09	−0.12	−0.11
**SO**_**2**_**(μg/m**^**3**^**)**	**0.02**	**0.93**	**0.93**	**0.92**	**0.95**	**1.01**	**1.06**
**NO**_**2**_**(μg/m**^**3**^**)**	**0.02**	0.11	0.11	0.10	0.12	0.18	0.20
**CO (μg/m**^**3**^**)**	**0.00**	0.02	0.02	0.02	0.02	0.02	0.02

Bold value indicates a significant value.

#### 3.3.2 Causality inference with a single treatment with a short-term lag

To eliminate the influence of different daily fluctuations and interactions between treatments, we used single-treatment causal inference models to explore the causality of each variable. By considering the different measurement units of different variables, we used causal effects in which multiply estimated effects with the IQR of the variables were used to evaluate the causality of each variable. This indicates the change in outcome when the treatment increased by one IQR. The estimated effects of these variables and the results of the three validation approaches are presented in Supplementary Tables S1 and S2, respectively. Table 3 presents the causal effects of the single-treatment causal inference models. As shown in Table 3, the causalities of both precipitation and air quality exhibited a short-term lag. Only in lag1 was it seen that precipitation had a significant causal effect on the DPAPVs (causal effect = 0.43, p < 0.05). However, AQI had significant causality only in lag3 (causal effect = 2.52, p < 0.05) and lag4 (causal effect = 2.52, p < 0.05). Visibility, SO_2_, NO_2_, and CO had relatively stronger causal effects on the DPAPVs, i.e., they had significant causal effects from lag0 to lag6. However, they exhibited maximal causal effects at different times: specifically, visibility in lag0 (causal effect = 4.78, p < 0.05), SO_2_ in lag5 (causal effect = 11.41, p < 0.05), NO_2_ in lag6 (causal effect = 6.90, p < 0.05), and CO in lag3 (causal effect = 10.67, p < 0.05). PM_2.5_ and PM_10_ had no significant causal effect on the DPAPVs at any time, a finding that was different from the results of the previous correlation analysis.

**Table 3:** The causal effect of each single treatment from lag0 to lag6.

	IQR	Lag0	Lag1	Lag2	Lag3	Lag4	Lag5	Lag6
**Precipitation (mm)**	3.6	0.32	**0.43**	0.32	0.11	0.00	0.22	0.11
**Visibility (m)**	4.6	**4.78**	**4.69**	**4.51**	**4.55**	**4.23**	**3.96**	**3.40**
**AQI**	42	1.68	1.26	1.68	**2.52**	**2.52**	1.68	2.10
**PM**_**2.5**_**(μg/m**^**3**^**)**	31	0.93	0.31	−0.62	−1.24	0.31	−0.62	−2.17
**PM**_**10**_**(μg/m**^**3**^**)**	49	1.47	1.96	1.47	−0.49	0.49	−1.47	−2.45
**SO**_**2**_**(μg/m**^**3**^**)**	7	**11.13**	**11.13**	**11.06**	**10.85**	**11.27**	**11.41**	**11.27**
**NO**_**2**_**(μg/m**^**3**^**)**	23	**5.75**	**5.06**	**5.29**	**5.52**	**5.75**	**6.44**	**6.90**
**CO (mg/m**^**3**^**)**	0.3	**9.42**	**9.93**	**10.57**	**10.67**	**9.79**	**8.56**	**8.18**

Bold value indicates a significant causal variable.

Although temperature, humidity, sea level pressure, and wind speed did not always have direct causal effects on DPAPVs, they did influence the treatments in the model. Therefore, we excluded these four indirect causes and explored the causal effects of the remaining variables. Fig. 3b presents an example of a model without indirect causes. In this model, only the treatment (CO) is indicated by an orange circle. The remaining variables were considered confounders. The orange arrows and lines indicate causality of the treatment.

The causal effect of each variable is shown in Table 4 (the estimated effect of these variables and the results of the three validation approaches are shown in Supplementary Tables S3 and S4, respectively). After adjusting for the effect of the four indirect causes, we noted that visibility, SO_2_, CO, and NO_2_ still had significant causal effects with the DPAPVs from lag0 to lag6. In addition, SO_2_ and CO had relatively high positive causal effects. These results indicate that these four variables were important causes of the DPAPVs, especially SO_2_ and CO. In contrast to the results in Table 3, AQI had no significant causality at any time. PM_2.5_ and PM_10_ had significant causal effects during lag6 and lag3–-lag6, which were not significantly different, as shown in Table 3. These results suggest that four indirect causes (humidity, temperature, sea level pressure, and wind speed) have a significant influence on the causality of AQI, PM_2.5_, and PM_10_. The effects of PM_2.5_ and PM_10_ in previous studies occurred primarily due to the combined effects of these interacting variables.

**Table 4:** The causal effect of immediate causal variables from lag0 to lag6 without indirect causes.

	IQR	Lag0	Lag1	Lag2	Lag3	Lag4	Lag5	Lag6
**Precipitation**	3.6	−0.14	0.14	0.25	0.14	0.29	0.29	**0.36**
**Visibility**	4.6	**4.78**	**4.69**	**4.51**	**4.55**	**4.23**	**4.00**	**3.40**
**AQI**	42	1.68	1.26	1.26	2.10	2.10	1.68	1.68
**PM**_**2.5**_	31	0.31	−0.31	−1.24	−2.17	−0.31	−1.24	**−3.10**
**PM**_**10**_	49	−1.47	−0.98	−1.96	**−3.43**	**−3.43**	**−5.88**	**−6.37**
**SO**_**2**_	7	**9.80**	**9.73**	**9.52**	**9.38**	**9.59**	**9.66**	**9.59**
**NO**_**2**_	23	**5.06**	**4.37**	**4.37**	**3.91**	**4.60**	**5.52**	**5.52**
**CO**	0.3	**9.40**	**9.89**	**10.47**	**10.57**	**9.60**	**8.42**	**7.98**

Bold value indicates a significant causal variable.

### 3.4 Causal effect of the improvement in air pollutants during eight years at hangzhou

**Fig. 4:**
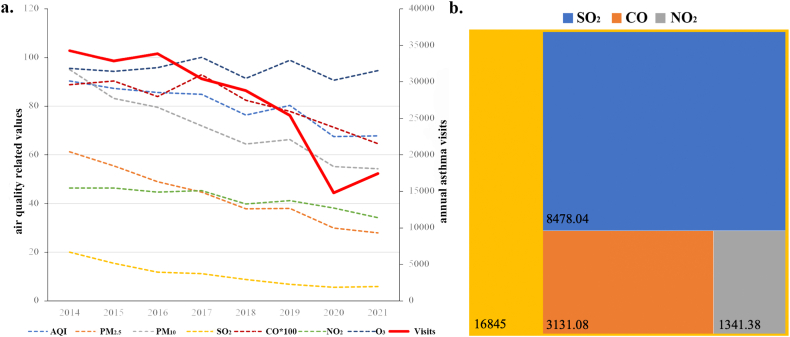
The quantitative causal effect between outdoor atmospheric quality and the pediatric asthma visits. a. The trend of air quality and annual asthma visits in Hangzhou from 2014 to 2021; b. Based on causal inference, the contribution of improvements in major air pollutants to the reduction in asthma visits in Hangzhou over an 8-year period was estimated. The area of the color block reflects the size of the contribution to reduce visits per year. The yellow color block in the background shows the true reduction in visits per year. (For interpretation of the references to color in this figure legend, the reader is referred to the Web version of this article.)

As shown in Fig. 4a, the overall number of asthma visits has decreased annually. The coronavirus disease 2019 pandemic, which began in the spring of 2020, also affected the number of respiratory disease visits, which can partially explain the rapid decline in 2020 [15]. The year-on-year reduction in atmospheric pollutants can explain this downward trend over the eight years of observation. Based on the estimated causal effect, we estimated that the improvements in major air pollutants in Hangzhou over the eight-year period contributed to the reduction of asthma visits. As shown in Fig. 4b, in total real reduction of 16,845 asthma visits in 2021 compared with 2014, the reduction in atmospheric SO_2_ concentration from 20.08 to 5.83 μg/m^3^ was ultimately estimated to reduce asthma visits by 8478.03 per year, which accounted for 50.3% of the overall reduction. During the eight-year period, the atmospheric CO concentration in Hangzhou decreased from 0.89 to 0.65 mg/m^3^, which ultimately resulted in an estimated 3131.08 fewer asthma visits per year. During the same period, atmospheric NO_2_ concentrations in the Hangzhou area decreased by 12.25 μg/m^3^, which ultimately resulted in an estimated 1341.38 fewer asthma visits per year. In short, data from Hangzhou demonstrated that improvement in air quality is effective in reducing pediatric asthma visits.

## 4 Discussion

Causal reasoning is an integral part of scientific inquiry, with a long history starting with ancient Greek philosophy. Estimating the causal effect seems straightforward; however, the fundamental challenge is that this calculation requires taking the difference between an observed outcome and a counterfactual explanation that we cannot observe. Given its importance, it is remarkable that many key statistical methods have been developed only during the last few decades [16,17]. “DoWhy” is a Python library that implements state-of-the-art causal inference methods. The key feature of DoWhy is its refutation API, which can automatically test causal assumptions for any estimation method, thereby making inferences more robust and accessible to non-experts.

Although there have been many association studies on asthma, this is the first causality analysis. The main results of this study were as follows. 1) SO_2_, NO_2_, CO, and visibility were exogenous causative variables of pediatric asthma visits, and they had maximum causality at different lag times. 2) When compared with other variables in this study, SO_2_ and CO were relatively more important pathogenic variables with stronger causal effects, reaching maxima at lag6 and lag3, respectively. 3) PM_2.5_ and PM_10_ were not independent exogenous causative variables of pediatric asthma visits. 4) The causal effect of air quality improvement on reducing pediatric asthma visits was quantitatively assessed for the first time in this study.

In this study, SO_2_ had the strongest positive causal effect on daily pediatric asthma patients. Similar studies have demonstrated a positive relationship between SO_2_ and pediatric asthma in China. A study in Xiamen, China showed that SO_2_ increased the incidence of uncontrolled asthma in children (OR = 1.252, P < 0.05) [18]. A study in Xian found that the relative risk (RR) of asthma in children was very sensitive to the change in SO_2_ concentration, which was 1.11 (95% CI: 1.02–1.21) for every 10 μg/m^3^ increase. Its RR was higher than those of the other variables and had a time lag effect (time lag of 5 d) [19]. In northern China, SO_2_ had a relatively high impact on pediatric asthma (RR = 1.17, 95% CI: 1.05–1.31), with a lag time of 5 days [20]. The difference in our results was the effect of short-term lag time, which may have been due to different locations being focused on during the other studies. It is plausible that SO_2_ exacerbates asthma. It has been reported that SO_2_ can promote airway responsiveness in a concentration-dependent manner by inducing local oxidative stress, which exacerbates asthma. This previous report also indicated that patients with asthma had a higher sensitivity to SO_2_ than to other air pollutants [21]. Overall, all of these studies support that SO_2_ has a greater impact on pediatric asthma and is an important pathogenic variable, which is consistent with the results of this study. The main contribution of this study is not only to confirm this correlation but also to quantitatively assess the causality of the change in visits that could be directly affected if SO_2_ concentrations were reduced.

We found that CO was another important causative variable in pediatric asthma, with a relatively high positive correlation and causality. The results of similar studies are consistent with those of the current one ours. In 2006, a study conducted in eight North American cities found that the 24-h average CO was a risk variable for asthma exacerbation in children (RR > 1) [22]. In 2014, research on children aged 3–10 years in New York found that an increase in the average CO in the previous 1–7 days was associated with an increase in the relative incidence of asthma visits in children [23]. In addition, a study in Padua (Italy) showed a significant positive association between outdoor CO concentration and lung function reduction in patients with asthma [24]. These conclusions suggest that our results in this study are reasonable, i.e., an increase in external CO concentration will lead to more pediatric asthma patients.

We observed that NO_2_ was an exogenous pathogenic variable in pediatric asthma, and this result is biologically plausible. It can enhance lung neutrophil inflammation and promote the Th2/Th17 phenotype, which increases morbidity in asthmatic individuals [25]. In addition, several epidemiological studies have demonstrated a sensitive relationship between the number of pediatric asthma visits and the concentration of NO_2_ [26,27].

We have also observed a positive causal effect of visibility on DPAPVs. This may be because patients prefer to go out during good weather. Therefore, a higher visibility may simply prompt more patients to visit the hospital rather than increase the total number of patients.

In contrast to other studies, in this study, PM_2.5_ and PM_10_ did not show DPAPVs causalities in the complete model, and their causalities were mainly inhibited by the combination of the other causal variables (SO_2_, CO, NO_2_, etc.) and indirectly causal variables (e.g., humidity, temperature, sea level pressure, and wind speed). In other words, simply controlling PM_2.5_ or PM_10_ does not necessarily reduce pediatric asthma visits. However, given their high correlation with SO_2_, CO, and NO_2_, PM_2.5_ and PM_10_ remain important parameters that reflect the impact of outdoor atmospheric quality and asthma visits.

This study had three major strengths. First, we applied a large pediatric asthma sample size (over 210,000) to identify exogenous variables causing pediatric asthma visits, which provided excellent statistical power. Second, this was the first study to explore the causality between pediatric asthma and external environmental variables (air pollutants and meteorological variables) using Dowhy causal inference models, the results of which are quantitative, making it helpful for discussing the dose-response relationship between these variables and pediatric asthma. Third, we discussed the effect of short-term lag and the interaction between external environment variables on the results of causal inference models.

Our study had four limitations. First, we assumed that each patient had the same exposure on the same day because the monitoring data from 24 monitoring stations in Hangzhou is similar and we represented the real-time exposure using daily mean meteorological data and daily mean air pollution data. Second, the risk variables for pediatric asthma vary (e.g., pollen, family inheritance, parental smoking history), as do the external environmental variables. However, these variables were excluded from this study, which may have caused a bias. Third, this study only explored the causal relationship between outdoor environmental variables and DPAPVs. Fourth, there was a limitation in the Dowhy causal inference model. The results of the model are based on a directed acyclic graph (DAG), which was built based on our knowledge. However, it is always difficult to appropriately select the relevant information to represent causal relationships.

## 5 Conclusions

The findings of this study enhance our understanding of the exogenous pathogenic variables of pediatric asthma in terms of environmental variables. The major findings of this study include the following: visibility, SO_2_, NO_2_, and CO have causal effects on the number of daily pediatric asthma patient visits for the short-term lag time; PM_2.5_ and PM_10_ did not have a significant causal effect with the influence of indirect causes; and SO_2_ and CO are the more important pathogenic variables, and their increase will lead to the largest increase in the number of daily asthma patients five days and three days later, respectively. Therefore, parents can strengthen the protection of children with asthma in the presence of high concentrations of SO_2_, CO, and NO_2_. Hospital administrators can schedule more doctors in the respiratory department after 3–5 days with higher SO_2_ and CO concentrations. Most importantly, continued improvements in air quality by government agencies, especially reductions in SO_2_, CO, and NO_2_ concentrations will reduce the need for asthma visits.

## 6 Ethics approval and consent to participate

This study was approved by the Institutional Review Board/Ethics Committee of the Children's Hospital, Zhejiang University School of Medicine (2018-IRB-046) and it was performed in accordance with the Declaration of Helsinki. The requirement for written informed consent was waived by the Institutional Review Board/Ethics Committee of the Children's Hospital, Zhejiang University School of Medicine, since the utilization of anonymized retrospective data does not require patient consent under local legislation.

## Author contribution statement

Yuqing Feng; Haomin Li; Xin Yang: Conceived and designed the experiments; Performed the experiments; Analyzed and interpreted the data; Wrote the paper.Yinshuo Wang; Lei Wu; Qiang Shu: Analyzed and interpreted the data; Contributed reagents, materials, analysis tools or data.

## Funding statement

This study was supported by the 10.13039/501100001809National Natural Science Foundation of China [82000030, 81871456].

## Data availability statement

Data will be made available on request.

## Declaration of competing interest

The authors declare that they have no known competing financial interests or personal relationships that could have appeared to influence the work reported in this paper.
