# Supplementary material for: Causal relationship between outdoor atmospheric quality and pediatric asthma visits in hangzhou
Source: Heliyon. 2023 Mar 7;9(3):e14271. doi: 10.1016/j.heliyon.2023.e14271 (PMC10023913; doi:10.1016/j.heliyon.2023.e14271)
Supplement: Multimedia component 1 [file mmc1.docx]

**Supplementary Material**

**
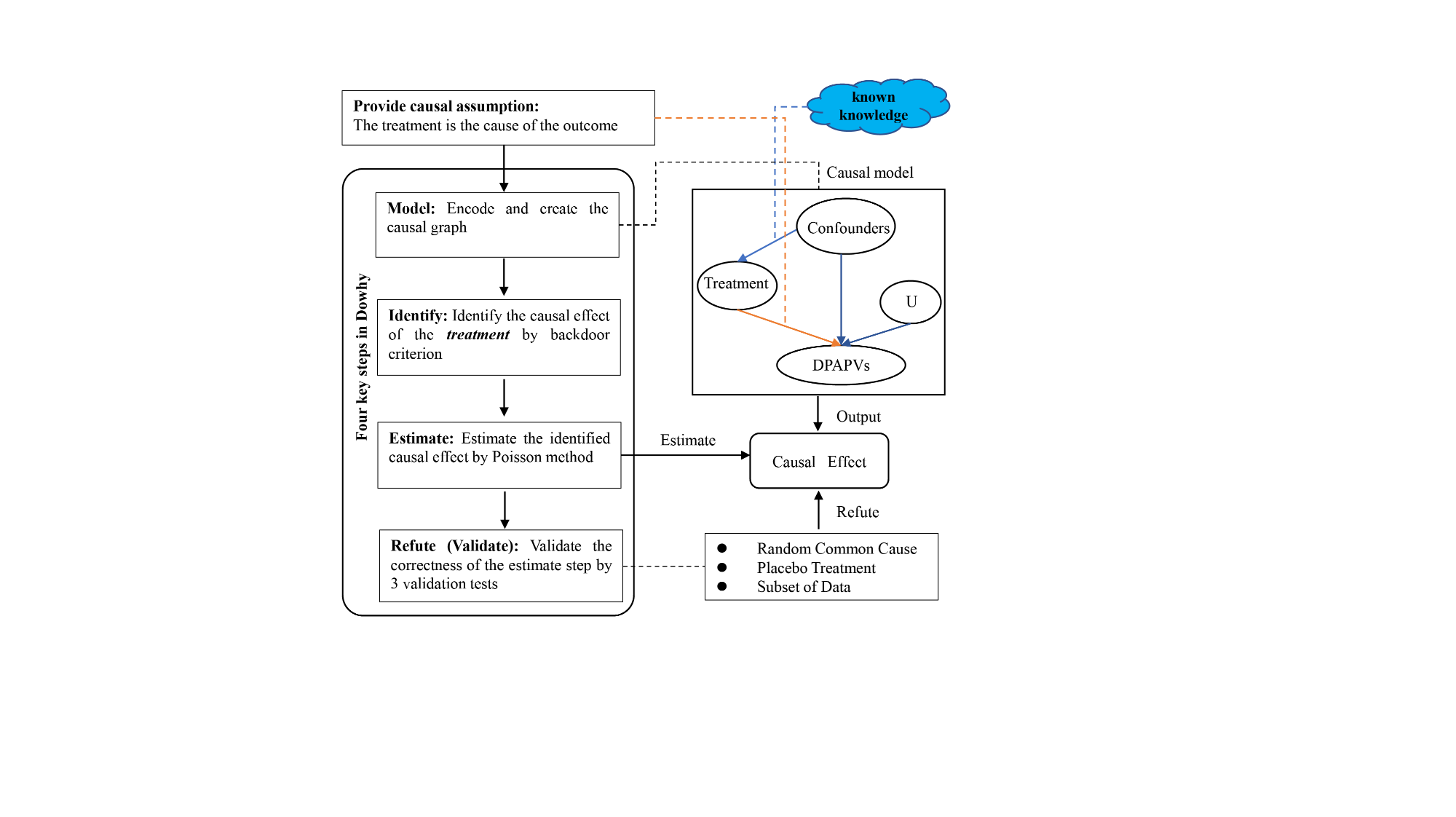
**

**Supplementary Fig. S1.** Steps of the causal inference model. U represented unobserved confounders.


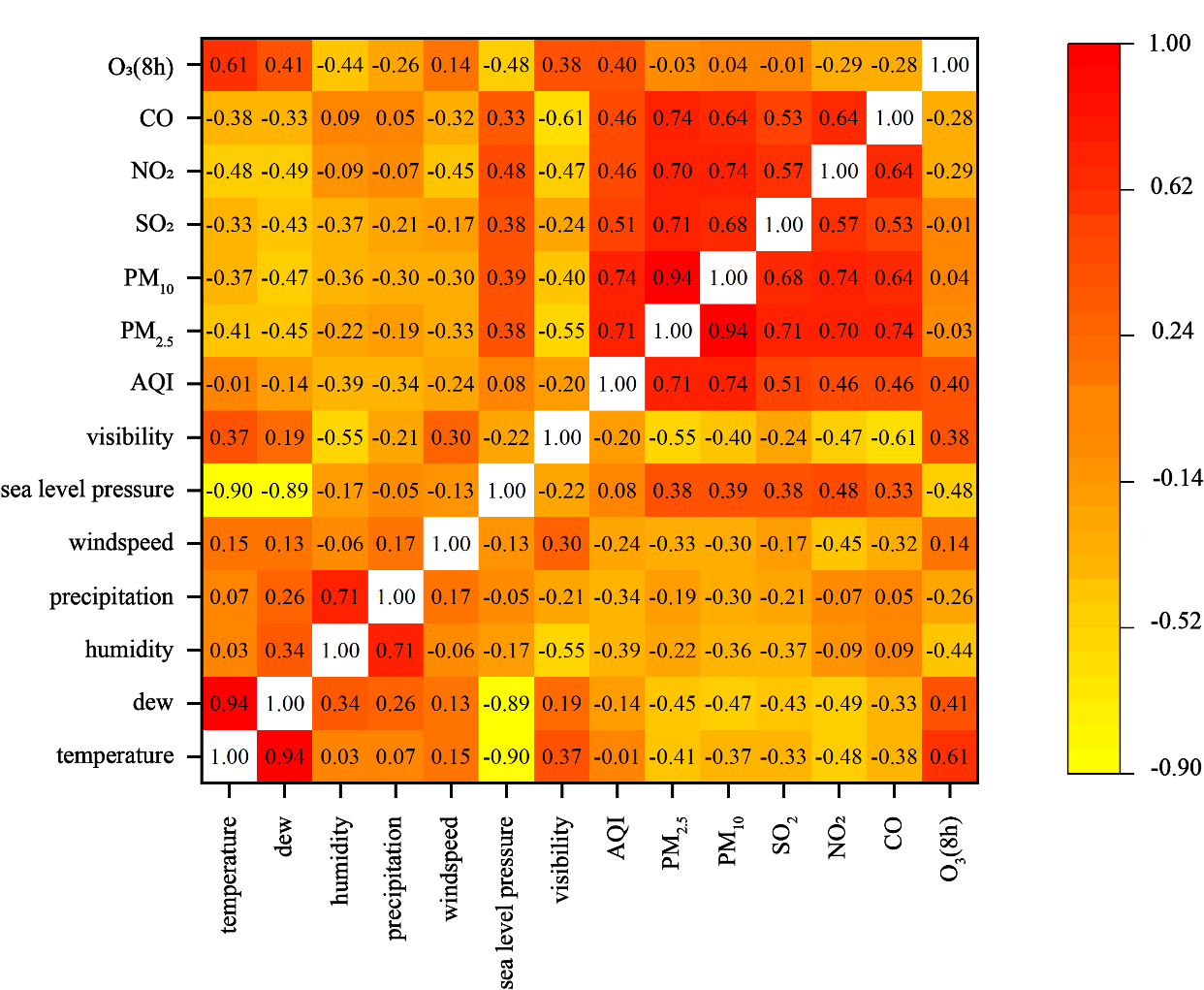


**Supplementary Fig. S2.** Spearman Correlation coefficients between daily air pollutant concentrations and meteorological variables in Hangzhou

**Supplementary Table S1.** The estimated effect between risk variables and DPAPV in single treatment model

|  | **Lag0** | **Lag1** | **Lag2** | **Lag3** | **Lag4** | **Lag5** | **Lag6** |
| --- | --- | --- | --- | --- | --- | --- | --- |
| **Precipitation** | 0.09 | 0.12* | 0.09 | 0.03 | 0.00 | 0.06 | 0.03 |
| **Visibility** | 1.04** | 1.02** | 0.98** | 0.99** | 0.92** | 0.86** | 0.74** |
| **AQI** | 0.04 | 0.03 | 0.04 | 0.06* | 0.06* | 0.04 | 0.05 |
| **PM_2.5_** | 0.03 | 0.01 | -0.02 | -0.04 | 0.01 | -0.02 | -0.07 |
| **PM_10_** | 0.03 | 0.04 | 0.03 | -0.01 | 0.01 | -0.03 | -0.05 |
| **SO_2_** | 1.59** | 1.59** | 1.58** | 1.55** | 1.61** | 1.63** | 1.61** |
| **NO_2_** | 0.25** | 0.22** | 0.23** | 0.24** | 0.25** | 0.28** | 0.30** |
| **CO** | 31.39** | 33.11** | 35.22** | 35.57** | 32.64** | 28.53** | 27.25** |

**Supplementary Table S2.** Results of three refute methods of significant causal variables in single treatment model from lag0 to lag6

| **Factor** | **Time** | **Estimated effect** | **Random common cause** | **Placebo treatment** | **Data subset** |
| --- | --- | --- | --- | --- | --- |
| **Precipitation** | Lag1 | 0.12 | 0.12 | 0.00 | 0.12 |
| **Visibility** | Lag0 | 1.04 | 1.04 | 0.00 | 1.05 |
|  | Lag1 | 1.02 | 1.02 | 0.00 | 1.02 |
|  | Lag2 | 0.98 | 0.98 | 0.00 | 0.97 |
|  | Lag3 | 0.99 | 0.99 | 0.00 | 0.98 |
|  | Lag4 | 0.92 | 0.92 | 0.00 | 0.91 |
|  | Lag5 | 0.87 | 0.87 | 0.00 | 0.88 |
|  | Lag6 | 0.74 | 0.74 | 0.00 | 0.73 |
| **AQI** | Lag3 | 0.06 | 0.06 | 0.00 | 0.06 |
|  | Lag4 | 0.06 | 0.06 | 0.00 | 0.06 |
| **SO_2_** | Lag0 | 1.59 | 1.59 | -0.02 | 1.59 |
|  | Lag1 | 1.59 | 1.59 | 0.00 | 1.59 |
|  | Lag2 | 1.57 | 1.58 | 0.00 | 1.59 |
|  | Lag3 | 1.55 | 1.55 | 0.00 | 1.56 |
|  | Lag4 | 1.61 | 1.61 | 0.00 | 1.62 |
|  | Lag5 | 1.63 | 1.63 | 0.00 | 1.63 |
|  | Lag6 | 1.61 | 1.61 | 0.00 | 1.62 |
| **NO_2_** | Lag0 | 0.25 | 0.25 | -0.02 | 0.25 |
|  | Lag1 | 0.22 | 0.22 | 0.00 | 0.22 |
|  | Lag2 | 0.23 | 0.23 | 0.00 | 0.24 |
|  | Lag3 | 0.24 | 0.24 | 0.00 | 0.25 |
|  | Lag4 | 0.25 | 0.25 | 0.00 | 0.25 |
|  | Lag5 | 0.28 | 0.28 | 0.00 | 0.28 |
|  | Lag6 | 0.30 | 0.30 | 0.00 | 0.31 |
| **CO** | Lag0 | 31.39 | 31.39 | 0.00 | 31.31 |
|  | Lag1 | 33.11 | 33.11 | 0.00 | 33.44 |
|  | Lag2 | 35.22 | 35.22 | 0.00 | 35.28 |
|  | Lag3 | 35.57 | 35.57 | 0.00 | 35.85 |
|  | Lag4 | 32.64 | 32.64 | 0.00 | 32.85 |
|  | Lag5 | 28.53 | 28.53 | 0.00 | 28.59 |
|  | Lag6 | 27.25 | 27.23 | 0.00 | 27.18 |

**Supplementary Table S3.** The estimated effect between risk variables and DPAPV in single treatment model (without indirect causes)

|  | **Lag0** | **Lag1** | **Lag2** | **Lag3** | **Lag4** | **Lag5** | **Lag6** |
| --- | --- | --- | --- | --- | --- | --- | --- |
| **Precipitation** | -0.04 | 0.04 | 0.07 | 0.04 | 0.08 | 0.08 | 0.10* |
| **Visibility** | 1.04** | 1.02** | 0.98** | 0.99** | 0.92** | 0.87** | 0.74** |
| **AQI** | 0.04 | 0.03 | 0.03 | 0.05 | 0.05 | 0.04 | 0.04 |
| **PM_2.5_** | 0.01 | -0.01 | -0.04 | -0.07 | -0.01 | -0.04 | -0.10* |
| **PM_10_** | -0.03 | -0.02 | -0.04 | -0.07* | -0.07* | -0.12** | -0.13** |
| **SO_2_** | 1.40** | 1.39** | 1.36** | 1.34** | 1.37** | 1.38** | 1.37** |
| **NO_2_** | 0.22** | 0.19** | 0.19** | 0.17** | 0.20** | 0.24** | 0.24** |
| **CO** | 31.32** | 32.96** | 34.89** | 35.22** | 31.99** | 28.06** | 26.59** |

**Supplementary Table S4.** Results of three refute methods of significant causal variables in single treatment model (without indirect causes) from lag0 to lag6

| **Factor** | **Time** | **Estimated effect** | **Random common cause** | **Placebo treatment** | **Data subset** |
| --- | --- | --- | --- | --- | --- |
| **Precipitation** | Lag6 | 0.10 | 0.10 | 0.00 | 0.10 |
| **Visibility** | Lag0 | 1.04 | 1.04 | 0.00 | 1.04 |
|  | Lag1 | 1.02 | 1.02 | 0.00 | 1.02 |
|  | Lag2 | 0.98 | 0.98 | 0.00 | 0.99 |
|  | Lag3 | 0.99 | 0.99 | 0.00 | 0.99 |
|  | Lag4 | 0.92 | 0.92 | 0.00 | 0.91 |
|  | Lag5 | 0.87 | 0.87 | 0.00 | 0.85 |
|  | Lag6 | 0.74 | 0.74 | 0.00 | 0.74 |
| **PM_2.5_** | Lag6 | -0.10 | -0.10 | 0.00 | -0.10 |
| **PM_10_** | Lag3 | -0.07 | -0.07 | 0.00 | -0.07 |
|  | Lag4 | -0.07 | -0.07 | 0.00 | -0.07 |
|  | Lag5 | -0.12 | -0.12 | 0.00 | -0.12 |
|  | Lag6 | -0.13 | -0.13 | 0.00 | -0.13 |
| **SO_2_** | Lag0 | 1.40 | 1.40 | -0.01 | 1.41 |
|  | Lag1 | 1.39 | 1.38 | 0.00 | 1.39 |
|  | Lag2 | 1.36 | 1.36 | 0.00 | 1.36 |
|  | Lag3 | 1.34 | 1.34 | 0.00 | 1.37 |
|  | Lag4 | 1.37 | 1.37 | 0.00 | 1.38 |
|  | Lag5 | 1.38 | 1.38 | 0.00 | 1.38 |
|  | Lag6 | 1.37 | 1.37 | 0.00 | 1.37 |
| **NO_2_** | Lag0 | 0.22 | 0.22 | -0.02 | 0.21 |
|  | Lag1 | 0.19 | 0.19 | 0.00 | 0.19 |
|  | Lag2 | 0.19 | 0.19 | 0.00 | 0.19 |
|  | Lag3 | 0.17 | 0.17 | 0.00 | 0.17 |
|  | Lag4 | 0.20 | 0.20 | 0.00 | 0.20 |
|  | Lag5 | 0.24 | 0.24 | 0.00 | 0.24 |
|  | Lag6 | 0.24 | 0.24 | 0.00 | 0.24 |
| **CO** | Lag0 | 31.32 | 31.31 | 0.00 | 31.45 |
|  | Lag1 | 32.96 | 32.96 | 0.00 | 32.93 |
|  | Lag2 | 34.89 | 34.89 | 0.00 | 35.08 |
|  | Lag3 | 35.22 | 35.22 | 0.00 | 35.22 |
|  | Lag4 | 31.99 | 32.00 | 0.00 | 32.07 |
|  | Lag5 | 28.06 | 28.05 | 0.00 | 28.51 |
|  | Lag6 | 26.59 | 26.59 | 0.00 | 26.60 |
